# The Somatic Aneuploidy Landscape of Adult Glia Reveals 16p as a Hotspot and Differentiates Mosaicism in Normal Glia from Chromosomal Instability in Glioblastoma

**DOI:** 10.21203/rs.3.rs-6497851/v1

**Published:** 2025-11-04

**Authors:** Cristina Montagna, Olivia Albert, Shixiang Sun, Jhih-Rong Lin, Moonsook Lee, Chang Chan, Alexander Maslov, Lisa Ellerby, Anita Huttner, Zhengdong Zhang, Jan Vijg

**Affiliations:** Rutgers Cancer Institute; Albert Einstein College of Medicine; Albert Einstein College of Medicine; Albert Einstein College of Medicine; Albert Einstein College of Medicine; Rutgers Institute of New Jersey; Albert Einstein College of Medicine; Buck Institute for Research on Aging; Yale University; Albert Einstein College of Medicine; Albert Einstein College of Medicine

**Keywords:** Aneuploidy, Single-nucleus whole-genome sequencing (snWGS), Non-neuronal glial cells, Glioblastoma (GBM), High-grade glioma, Tumor heterogeneity, Loss of chromosome X (LoX), Chromosome 16p11.2 CNV

## Abstract

Aneuploidy, an abnormal number of chromosomes, is a hallmark of cancer and has been proposed as an initiating event in tumorigenesis. In glioblastoma (GBM), a highly aggressive brain tumor, cells almost universally display gain of chromosome 7 and loss of chromosome 10. However, it remains unclear whether these alterations arise *de novo* during malignant transformation or reflect pre-existing chromosomal instability in normal brain tissue. Here, we used single-nucleus whole-genome sequencing (snWGS) on 225 NeuN-negative (non-neuronal) cortical nuclei from 12 healthy individuals and 6 GBM patients, including matched tumor cores and non-tumor brain regions. In healthy brains, approximately 15% of glial nuclei harbored somatic aneuploidies, most often involving chromosome arms, with recurrent 16p alterations detected in up to 3% of nuclei from both healthy controls and GBM non-tumor tissue. These findings establish 16p is a hotspot of structural variation in adult glia. Non-tumor regions in GBM patients closely resembled healthy controls in aneuploidy burden and chromosomal instability metrics and lacked hallmark tumor alterations. In contrast, GBM tumors exhibited significantly elevated aneuploidy (~50%), enrichment for canonical chromosomal instability-driven events, and sex-specific karyotype patterns, consistent with transformation-associated chromosomal instability. Thus, aneuploidy is a recurrent but constrained feature of normal adult glia, whereas chromosome instability and GBM-defining aneuploidies emerge only during malignant transformation.

## Introduction

Aneuploidy, defined as the gain or loss of whole chromosomes or chromosome arms, is among the most pervasive genomic alterations in human cancer (1). It is detected in nearly all solid tumors and often arises early in tumorigenesis (2–4). Unlike point mutations that affect individual genes, aneuploidy alters the dosage of hundreds of genes at once, disrupting transcription, protein homeostasis, and metabolism (5–7). While generally considered detrimental in normal cells (5, 6, 8), specific aneuploidies can provide adaptive advantages in tumors, promoting clonal expansion under selective pressure such as hypoxia, immune surveillance, and anticancer therapy (9, 10). In some contexts, tumor cells may even become addicted to aneuploidy, in a manner analogous to oncogene addiction (11).

Evidence from genetically engineered mouse models further underscores the requirement of aneuploidy in malignant evolution: even when tumor initiation is driven by strong oncogenes or tumor suppressor loss, non-random chromosomal alterations emerge that mirror those in human cancers (12–15). These recurrent, cancer-type-specific patterns suggest that aneuploidy is not merely tolerated but actively selected, reshaping cellular physiology in context-specific ways. For example, gain of chromosome 3 is common in squamous carcinomas (16), whereas glioblastomas (GBM) almost universally display gain of chromosome 7 and loss of chromosome 10 (17).

Although aneuploidy is a defining hallmark of malignant tissues, it is not restricted to them. Low-frequency somatic aneuploidies have been reported in normal tissues, including breast (18) and brain (19–22), and their prevalence increases with age (19, 23, 24). This has led to the hypothesis that specific chromosomal imbalances might prime tissues for malignant transformation (25–27). In this model, cells that acquire canonical tumor-associated aneuploidies in otherwise normal tissue could gain a fitness advantage that lowers the barrier to transformation once driver point mutations occur, helping explain why most cancers require multiple genetic hits (28). In some contexts, point mutations may initiate transformation and aneuploidy subsequently provide a permissive state that favors progression; in others, early aneuploidy may create selective pressures that influence which mutations are retained and expanded. Because most aneuploidies are deleterious, only a restricted set persist, acting as filters that define the recurrent, tissue-specific karyotypes observed in human cancers.

Mechanistically, aneuploidy arises from chromosomal instability (CIN), the persistent missegregation of chromosomes during mitosis. CIN is a consequence of spindle checkpoint failure, merotelic attachments, cohesion defects, or replication stress (29, 30). Aneuploidy represents the structural outcome of these processes. Together, CIN and aneuploidy establish a cycle in which ongoing missegregation produces karyotypic diversity, from which selective pressures favor aggressive sub clones (29). In this way, CIN-driven aneuploidy promotes clonal selection and tumor adaptation. While aneuploidy is a hallmark of advanced malignancies, the timing of CIN onset during tumor initiation remains unresolved. In some tissues such as Barrett’s esophagus and colorectal adenomas, CIN emerges early and acts as a field defect (3, 31).

This concept of field cancerization was first described in squamous epithelium (32) and later reinterpreted in evolutionary terms (33). It highlights how genomic instability can extend across histologically normal tissue. By contrast, in other tissues, such as breast and lung, CIN appears restricted to malignant cells, arising only after enabling events such as checkpoint loss or whole-genome duplication (WGD) allow tolerance of widespread chromosomal imbalance (10, 13, 34). Thus, the role and timing of CIN may be tissue-specific, with some tissues manifesting CIN early and others only after tumor-promoting adaptations are acquired.

GBM provides a powerful system to address this question. GBM is an aggressive glial progenitor–derived brain tumor with a median survival of 12–18 months. It is characterized by extensive CIN and widespread aneuploidy, with ~ 80–90% of cases harboring the highly recurrent gain of chromosome 7 and loss of chromosome 10 (+7/–10) signature, which amplifies oncogenes such as *EGFR*, *MET*, and *CDK6* while deleting tumor suppressors including *PTEN* and *MGMT* (17). WGD, also frequent in GBM, is increased with aneuploidy burden and may buffer copy-number imbalances, enabling tolerance of further chromosomal remodeling (34, 35). The recurrence of +7/–10 strongly suggests it is an early clonal driver, establishing a permissive genomic landscape for subsequent diversification. Yet it remains unresolved whether such hallmark aneuploidies originate in normal glial cells and persist until malignant transformation, or whether they arise only after tumor initiation, once checkpoint attenuation and WGD enable tolerance of CIN. Epidemiologically, GBM incidence is ~ 1.6-fold higher in men than women (36, 37), and recent multi-omics analyses have revealed sex-biased genomic features (38), raising the possibility that selective pressures shaping CIN and aneuploidy may also differ by sex.

Importantly, while aneuploidy can be detected as recurrent and clonal in tumors, CIN itself is far more difficult to measure. Missegregation events are stochastic, vary from cell to cell, and rarely manifest as a stable clonal signature. Therefore, CIN is best inferred at single-cell resolution, and its prevalence in non-tumor tissues of cancer patients remains largely unknown (15, 39). This distinction is mechanistically significant. If non-tumor glia in GBM patients already carry elevated or GBM-like aneuploidies, then CIN can function as a priming event, similar to Barrett’s esophagus or colon adenomas where aneuploidy predicts malignant progression. If instead non-tumor glia resembles healthy controls, then CIN represents a transformation-associated state confined to the malignant compartment. In the latter case, aneuploidy should be viewed not as a predisposing event but as an adaptive mechanism that accelerates tumor evolution.

Outside of cancer, somatic aneuploidy has also been linked to aging, environmental stress, and neurodegeneration. Early fluorescence *in situ* hybridization (FISH) studies reported high rates of neuronal aneuploidy, particularly in aged or diseased brain (21, 22, 40), but more recent single-cell sequencing analyses have consistently found lower frequencies (20, 41). These discrepancies likely reflect methodological differences, sampling biases, or cohort heterogeneity (42). Importantly, while neurons have been the main focus of brain aneuploidy studies, glial cells remain comparatively understudied. Unlike postmitotic neurons, glia retain proliferative potential throughout life, making them intrinsically more susceptible to mitotic errors and chromosomal missegregation (43). Large CNVs further contribute to somatic mosaicism in neurons (44–46), but whether similar structural variation occurs in glia, and if so, whether it follows recurrent or tissue-specific patterns, remains unknown.

Evidence from other tissues shows that aneuploidy can precede malignant transformation: aneuploid clones have been detected in premalignant lesions of the colon (47), gastrointestinal tract (26), cervix (48), ovary (49), and breast (18). Likewise, somatic driver mutations were found in histologically normal skin, colon, and lung (50–53). These field effects raise the possibility that normal glia in GBM patients might already carry tumor-relevant aneuploidies. Although dosage interactions between chromosome 7 gain and chromosome 10 loss have been observed in glial precursors (54), whether such alterations exist in histologically normal brain tissue remains unresolved.

Here, we address this gap by performing single-nucleus whole-genome sequencing (snWGS) (55, 56) of NeuN-negative nuclei from adult cortex in three cohorts: disease-free individuals, GBM tumor cores, and matched non-tumor brain regions distal to the tumor. This design enables high-resolution profiling of whole-chromosome and segmental aneuploidy, structural heterogeneity, and ploidy states in glial cells across normal, tumor-adjacent, and malignant compartments. We show that somatic aneuploidy is a recurrent feature of adult human glial cells and identify chromosome 16p as a frequently altered hotspot with potential biological relevance beyond cancer. Importantly, the alterations in non-tumor glia arise through mechanisms distinct from the CIN-driven imbalances that define GBM, providing new insight into the origins and tissue specificity of aneuploidy.

## Results

### Somatic Aneuploidy Defines the Genomic Landscape of Adult Human Glial Cells

**Figure 1:**
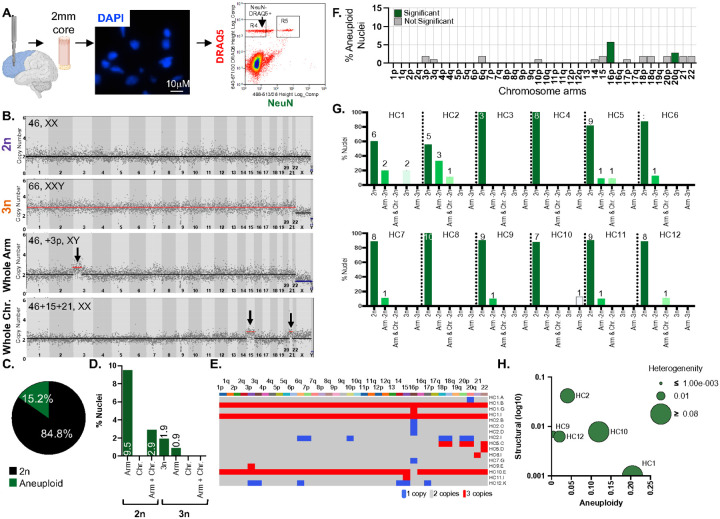
Chromosomal aneuploidies in NeuN-neg cells of the aged human cortex. **A.** Single NeuN-neg nuclei isolation workflow. A 2mm biopsy punch core was extracted from frozen brain tissue, homogenized, and stained with NeuN. DRAQ5 was used to differentiate nuclei from debris. NeuN-neg nuclei were isolated into single tubes using fluorescence activated cell sorting (FACS) (R4 in FACS plot). **B.** Representative single cell profiles: a euploid nucleus (2n), a triploid nucleus (3n), a nucleus with a whole arm aneuploidy (Whole Arm), and a nucleus with whole chromosome aneuploidies (Whole Chr.). The X-axis represents the chromosomes; the Y-axis indicates the inferred copy number based on read coverage. Each dot corresponds to the copy number of a 5Mb bin of reads generated by Ginkgo. Blue, black, and red lines represent one, two, and three copies, respectively. **C.** Pie chart showing the percentage of euploid (black) and aneuploid (green) nuclei in healthy controls. **D.** Bar chart categorizing aneuploidy types in healthy controls: 2n with an aneuploid arm, 2n with an aneuploid chromosome, 2n with both arm and chromosome aneuploidies, 3n without aneuploidies, and 3n with aneuploid arms. **E.** Heatmap summarizing aneuploidies in all healthy control nuclei. Nuclei without aneuploidies were excluded. X-axis shows chromosomes; Y-axis shows nuclei with aneuploidies. Blue, grey, and red boxes represent one, two, and three copies, respectively. **F.** Bar graph depicting the percentage of aneuploid nuclei for each chromosome arm for all healthy control nuclei (n=105). Green bars indicate chromosomes which were significantly more aneuploid in HCs than other chromosomes in gray. **G.** Bar graph depicting the percentage (Y-axis) and number (above the bars) of aneuploid nuclei detected in each healthy control, classified by aneuploidy type (chromosome arm, whole chromosome) and ploidy background (2n or 3n). **H.** Bubble plot illustrating chromosomal instability metrics for healthy controls. Y-axis: structural score (changes in ploidy by breakpoints); X-axis: aneuploidy score (amount of chromosome gain or loss in single nuclei); bubble size: heterogeneity score (variability of aneuploidies between cells). HCs 3,4,6,7,8 not included as their scores were 0 for all three measures.

To assess aneuploidy in non-neuronal nuclei of the adult, normal human brain, we analyzed cortical tissue from 12 disease-free individuals (aged 51.8±13.8 years, Table 1) using low-coverage single nucleus whole-genome sequencing (snWGS). Nuclei were isolated from frozen brain samples using Fluorescence-Activated Cell Sorting (FACS) following NeuN and DRAQ5 staining (Figure 1A). This strategy enabled the detection of euploid (2n), polyploid (3n), and aneuploid nuclei, including chromosome arm and whole-chromosome alterations (Figure 1B). Methodological accuracy to detect expected karyotypes was confirmed using euploid and Trisomy 21 (T21) control cell lines (**Supplementary Figure 1, Supplementary Tables 1–2**).

Autosomes were the primary focus of the initial analysis, as sex chromosomes were assessed separately due to their unique structural properties and technical challenges in sequence read alignment, particularly the Y chromosome, which is highly enriched in repetitive and low-complexity regions that complicate accurate copy number estimation in ultra-low coverage single-cell whole genome sequencing (57). The mean sequencing depth for healthy control nuclei (HC) was 0.15 ± 0.13X (**Supplementary Tables 3**, **Supplementary Figure 2A**), which was not significantly different from euploid and T21 control nuclei (one-way ANOVA, p = 0.19, n.s.).

Among 105 NeuN-neg HC nuclei, 89 (84.8%) were euploid and 16 (15.2%) were aneuploid (Figure 1C and **Supplementary Table 4**). Of the aneuploid nuclei, 10 (9.5%) carried chromosome arm aneuploidies, 1 (0.9%) showed an arm-level change in a triploid background, and 3 (2.9%) contained both arm and chromosome-level alterations in a diploid background. Two nuclei (1.9%) had a triploid karyotype without additional detectable aneuploidies (Figure 1D–E). Autosomal gains and losses occurred at similar frequencies, accounting for 10 (35.7%) and 18 (64.3%) aneuploidies respectively (p=0.094, chi-square test) (**Supplementary Figure 2B**). Aneuploid nuclei exclusively exhibited either gains or losses, never both (**Supplementary Figure 2C**), with no significant chromosome-specific bias in gains versus losses (p=0.81) (**Supplementary Figure 2D**).

In healthy controls, 24 chromosome arms exhibited no aneuploidy, while 12 arms (3p, 3q, 6q, 10p, 14, 15, 17p, 18p, 18q, 20p, 21, and 22) showed aneuploidy in ≤2% of nuclei. Chromosomes 16p and 20q displayed significantly higher aneuploidy frequencies compared to random chance (16p: p=1.21×10^−4^, 20q: p=**0.042)** (Figure 1F).

The percentage of aneuploid nuclei per individual ranged from 0 to 44% (**Supplementary Figure 2E**), with no significant difference in overall genome alterations **(Supplementary Figure 2F)**. This broad range in aneuploidy burden suggests substantial inter-individual variability in glial genome stability. Importantly, individuals carrying aneuploid nuclei showed a significantly greater number of affected chromosomes per nucleus and a lower proportion of euploid cells than individuals without detectable aneuploidy (W = 25.5, p = 0.024; W = 27, p = 0.0095). These differences indicate that the variation we observed reflects genuine biological heterogeneity across subjects rather than random sampling noise (Figure 1G).

To further investigate the extent and pattern of chromosomal instability (CIN) in these non-neuronal glial cells, we applied three metrics: structural score (number of ploidy changes per chromosome), aneuploidy score (number of gained or lost chromosomes per subject), and heterogeneity score (variability of aneuploidies among nuclei from the same subject). Aneuploidy scores were similar across most individuals, except for HC1 and HC10, which had higher scores due to the presence of 3n nuclei (two in HC1, one in HC10). Individuals with scores near zero were excluded from the plot (Figure 1H). In this plot, the x-axis shows the fraction of aneuploid nuclei in each NT sample, the y-axis structural complexity (log_10_ scale), and the bubble size karyotypic heterogeneity.

### CIN-Driven Aneuploidies and Clonal Architecture Distinguish Glioblastoma Tumors

We next analyzed both tumor (TUM) and non-tumor (NT) brain regions from six GBM subjects (mean age 63 ± 5.8) (Table 2). The average sequencing depth was 0.21 ± 0.34 for TUM and 0.13 ± 0.07 for NT (**Supplementary Table 5** and **Supplementary Figure 3A**), which did not differ significantly from each other, form HC, of form euploid and T21 control datasets (one-way ANOVA, p = 0.10, n.s.).

**Figure 2:**
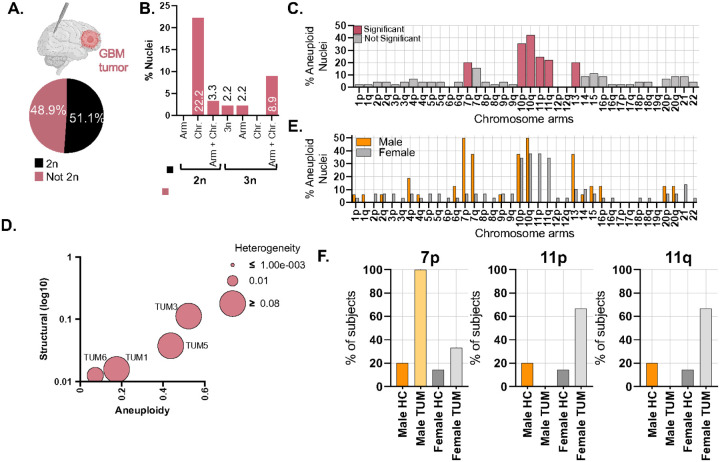
Tumor exhibits highest aneuploidy frequencies, significantly different from both NT-D and HCs. **A.** Pie chart showing the percentage of euploid (black) and aneuploid (pink) nuclei in tumors. **B.** Bar chart categorizing aneuploidy types in TUM: 2n with an aneuploid arm, 2n with an aneuploid chromosome, 2n with both arm and chromosome aneuploidies, 3n without aneuploidies, and 3n with aneuploid arms. **C.** Bar graph depicting the percentage of aneuploid nuclei for each chromosome arm for all TUM nuclei (n=45). Chromosome arms significantly more aneuploid are in pink, not, in gray. **D.** Bubble plot illustrating chromosomal instability metrics for TUM. Y-axis: structural score (changes in ploidy by breakpoints); X-axis: aneuploidy score (amount of chromosome gain or loss in single nuclei); bubble size: heterogeneity score (variability of aneuploidies between cells). **E**. Percentage of aneuploid nuclei for all tumors sequenced, separated by gender. Males in orange, females in grey. **F.** Chromosomes which had a significant difference between male and female tumors, chromosome 7p as well as 11p and 11q. Male and female tumors are also compared to healthy controls. 7p: Male HC v Male TUM (p=<0.0001); Male TUM v Female TUM (p=<0.0001), 11p: Female HC v Female TUM (p=0.0236); Male TUM v Female TUM (p=0.0599), 11q: Male TUM v Female TUM (p=0.0475), Female HC v Female TUM (p=0.0178).

Among 45 TUM nuclei, 23 (51.1%) were euploid, and 22 (48.9%) were aneuploid (Figure 2A). Aneuploid nuclei exhibited diverse karyotypic alterations: 10 (22.2%) showed chromosome-level aneuploidies; 6 (13.3%) harbored combined arm- and chromosome-level aneuploidies; 1 (2.2%) was triploid without detectable aneuploidies; 1 (2.2%) had an arm-level aneuploidy in a triploid background; and 4 (8.9%) exhibited both arm- and chromosome-level aneuploidies in polyploid backgrounds (two in a 3n background and two in either a 4n or 5n background) (Figure 2B).

Aneuploidy was not randomly distributed across the genome. Most chromosome arms showed aneuploidy in ≤11% of nuclei, including 1p, 1q, 2p, 2q, 3p, 3q, 4p, 4q, 5p, 5q, 6q, 8p, 8q, 9p, 9q, 14, 15, 16p, 16q, 17p, 17q, 18p, 18q, 20p, 20q, 21 and 22. In contrast, chromosome arms 7p, 10p, 10q, 11p, 11q, and 13 were significantly more aneuploid than expected by random chance (p-values: 9.77 × 10^−3^ for 7p, 2.63 × 10^−6^ for 10p, <2.63 × 10^−6^ for 10q, 7.89 × 10^−4^ for 11p, 2.96 × 10^−3^ for 11q, and 9.77 × 10^−3^ for chromosome 13) (Figure 2C). Across all tumor nuclei, we detected 140 aneuploidies, comprising 35 (25%) chromosome arm gains and 105 (75%) losses, with no significant bias toward gains or losses (p=0.0922) (**Supplementary Figure 3C,D**). Chromosome instability metrics varied substantial between subjects, consistent with inter-patient heterogeneity in tumor karyotype evolution (Figure 2D).

We also evaluated sex-specific differences in tumor aneuploidy. GBM incidence is approximately 1.6-fold higher in men than in women (36,37). In our cohort, nuclei from male tumors more frequently harbored 7p aneuploidy (p<0.0001), while 11p and 11q were more frequently altered in female tumors (p=0.0599 and p=0.0475, respectively) (Figure 2E–F). These differences suggest potential sex-biased patterns of chromosomal alterations in GBM.

Finally, we assessed the relationship between aneuploidy and whole-genome duplication (WGD), a common feature of high-grade gliomas (34,35). WGD was significantly associated with aneuploidy (p=0.0013), with WGD nuclei being 18 times more likely to be aneuploid than diploid nuclei (odds ratio: 18.08; 95% CI: 2.15–849.90; **Supplementary Table 7**).

### Recurrent 16p Alterations Reveal a Non-Tumor-Specific Hotspot of Somatic Variation

**Figure 3:**
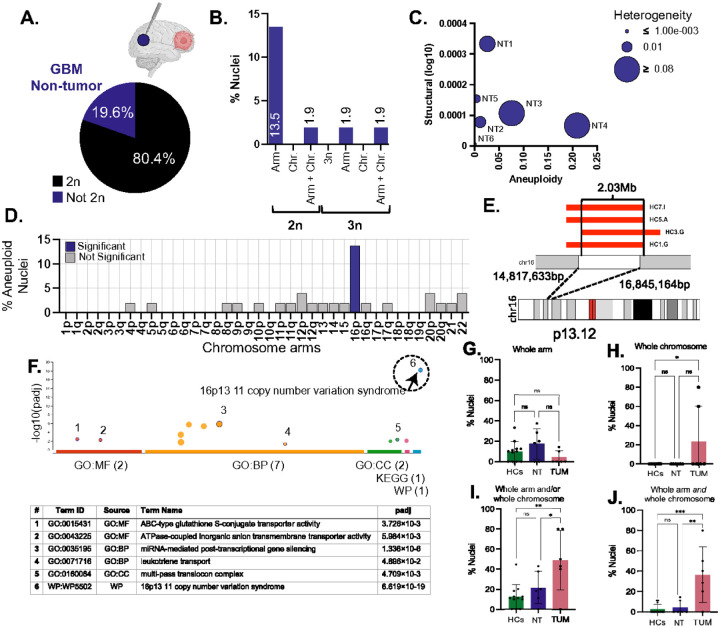
Non-tumor distal tissue of GBM patients displays low aneuploidy frequency. 16p aneuploidies emerge as a genomic signature in disease-free brain. **A.** Pie chart showing the percentage of euploid (black) and aneuploid (purple) nuclei in NT-D cohort. **B.**Bar chart categorizing aneuploidy types in NT-D: 2n with an aneuploid arm, 2n with an aneuploid chromosome, 2n with both arm and chromosome aneuploidies, 3n without aneuploidies, and 3n with aneuploid arms. **C.** Bubble plot illustrating chromosomal instability metrics for NT-D. Y-axis: structural score (changes in ploidy by breakpoints); X-axis: aneuploidy score (amount of chromosome gain or loss in single nuclei); bubble size: heterogeneity score (variability of aneuploidies between cells). **D.** Bar graph depicting the percentage of aneuploid nuclei for each chromosome arm for all NT-D nuclei (n=51). Chromosome arms significantly more aneuploid are in purple, not, in gray. **E.** Map of chromosome 16p, showing specific 2.03 Mb region of aneuploidy overlap between the four healthy control individuals. **F.** Gene Ontology (GO) analysis of region on chromosome 16p in D. Y-axis is adjusted p-value, x-axis is the source of the significant GO terms (also in chart). 16p13.11 CNV syndrome being the most significant. **G. H. I, J.** Bar charts comparing the percentage of nuclei with aneuploidies (y-axis) across three groups (xaxis), categorized by aneuploidy type (whole arm, whole chromosome, whole arm and/or whole chromosome and whole arm *and* whole chromosome). One-way ANOVA p-values are indicated: whole chromosome (HCs v TUM p=0.0419) whole arm and/or chromosome (HCs v TUM p=0.0023, NT-D v TUM p=0.0499); whole arm *and* whole chromosome (HCs v TUM p=0.0003, NT-D v TUM p=0.0021).

In NT brain regions form GB patients, 41 of 51 Neu-N-negative nuclei (80.4%) were euploid and 10 (19.6%) were aneuploid (Figure 3A). Among the aneuploid nuclei, 7 (13.5%) harbored arm-level alterations; 1 (1.9%) displayed combined arm- and chromosome-level changes in a diploid background; 1 (1.9%) had arm-level aneuploidy in a triploid nucleus; and 1 (1.9%) exhibited both types of alterations in a triploid background (Figure 3B).

Across samples, NT nuclei exhibited uniformly low structural complexity, clustering along the lower portion of Figure 3C. While structural complexity was consistently low, aneuploidy burden varied across NT samples, ranging from minimal to levels approaching those seen in tumors. Genomic profiles in NT and TUM nuclei differed significantly based on Euclidean distance (p = 0.03), underscoring the genomic divergence between these compartments (**Supplementary Figure 3H**).

Across the 51 NT nuclei, 19 chromosome arms showed no detectable alterations. Other 18 arms including 4p, 5p, 8q, 9p, 10p, 11p, 11q, 12p, 12q, 13, 14, 15, 16q, 17q, 20p, 20q, 21, and 22, were altered in fewer than 5% of nuclei. In contrast, chromosome arm 16p was detected in 14% of NT nuclei, a significantly elevated frequency compared to random expectation (p = 7.89 × 10^−6^, Figure 3D). Unlike the CIN-driven patterns observed in tumors, NT nuclei displayed a distinct, recurrent involvement of 16p, suggesting a locus-specific susceptibility unrelated to malignancy.

To investigate this further, we examined whether 16p harbored focal structural alterations in addition to whole-arm changes. The 16p13.11 region is known for its germline instability and has been associated with multiple neurodevelopmental phenotypes, raising the possibility that somatic variation at this locus may also contribute to glial heterogeneity (58).

We identified a segmental loss involving 16p13.11 in four nuclei, one each from HC1, HC3, HC5, and HC7, with a minimal overlapping region of 2.03 Mb (chr16:14,817,633–16,845,164) (Figure 3E). Nuclei from HC1, HC5, and HC7 shared a 2.53 Mb deletion spanning chr16:14,310,885–16,845,164, while HC3 exhibited a distinct 2.53 Mb deletion extending from chr16:14,817,633–17,356,860. This interval includes 73 coding transcripts representing ~30 unique genes, many of which are expressed in the brain.

Gene enrichment analysis revealed significant association with the 16p13.11 copy number variation syndrome pathway (WP5502; adj. p=4.888×10^−19^) (Figure 3F), a locus recurrently disrupted in neurodevelopmental disorders such as autism, intellectual disability, epilepsy, schizophrenia, and Attention-deficit/hyperactivity disorder (ADHD) (59).

Beyond 16p, no other loci shoed evidence of recurrent or tumor-associated CIN patterns in NT regions, reinforcing the genomic distinction between non-tumor and malignant compartments. Of all aneuploidies observed in NT nuclei, 5 (17.8%) were gains and 23 (82.4%) were losses. Each nucleus exhibited either gains or losses; only one nucleus displayed both (**Supplementary Figure 3E-G**).

### Non-Tumor Glial Cells Lack CIN Signatures or GBM-Defining Aneuploidies

We first compared aneuploidy frequencies between healthy controls and non-tumor glial nuclei. No significant differences were observed in the prevalence of whole-arm or whole-chromosome aneuploidies between these groups (Figure 3G,H). Likewise, no significant differences in CIN metrics or ANCA scores were found, further confirming that non-tumor glial cells show no evidence of tumor-associated genomic instability.

In contrast, tumors nuclei showed significantly higher frequency of whole-chromosome aneuploidy than HCs (p=0.0023; Figure 3H). Nuclei carrying either arm- or chromosome-level alterations were also significantly more common in tumors than compared to both HCs (p=0.0023) and NT regions (p=0.0499; Figure 3I). Nuclei with both types of alterations were enriched in tumors relative to HCs (p=0.0003) and NT (p=0.0021; Figure 3J).

**Figure 4:**
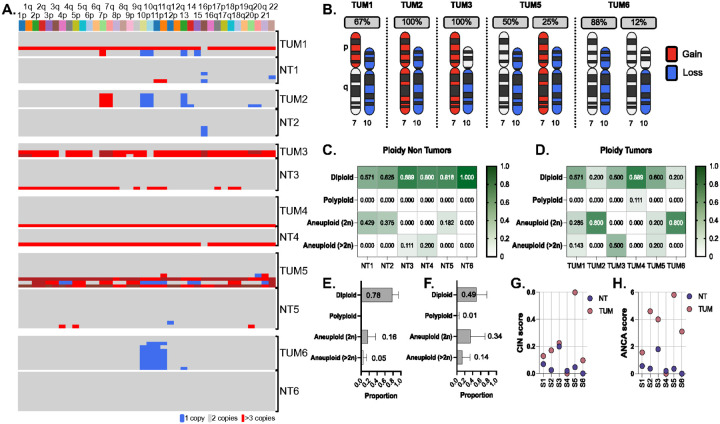
Clonal aneuploidies defining the genomic landscape of GBM are absent in matching non-tumor tissues. **A.**Heatmaps summarizing aneuploidy profiles for matched GBM tumor and non-tumor distal nuclei. Each row corresponds to one nucleus, with the thin white lines separating matched tumor and non-tumor nuclei and thick white line separates the GBM patients. Blue indicates one copy, gray indicates two copies, red indicates three copies and brick red indicates four copies. **B.** Every tumor with aneuploidies represented, focused on the p and q arms of chromosomes 7 and 10. Percentage indicates how many of the aneuploid nuclei in each individual presents with the highlighted aneuploidy. Arms in red are gained, black and white are euploid and blue is lost. Tumors with multiple aneuploidy combinations of chromosomes 7 and 10 are represented by two sets of chromosomes, with their percentages representing the frequency of these aneuploidies in the aneuploid presenting nuclei. **C,D.** Heatmaps highlighting the ploidies present in NT-D (C) and TUM (D). Y-axis represents different ploidies Diploid being a normal chromosome count, polyploid meaning multiple copies of a set of chromosomes, aneuploid (2n) represents nuclei with a normal 2n modal copy number, but aneuploidies of one or a few chromosomes and finally aneuploid (>2n) which indicates cells with multiple copies of a set of chromosomes AND additional aneuploidies; x-axis is each individual. Shade of green represents frequency of ploidy state. **E.F.** Summary plots of C and D, combining all NT-D subjects (E) and all TUM subjects (F). Y-axis indicates the same ploidy states as C and D. X-axis is the proportion of nuclei for all NT-D (E) and TUM (F) nuclei presenting with each ploidy state. **G.** Plot representing CIN score for NT-D and TUM. Along the Y-axis is the CIN score (chromosomal instability), along the x-axis is each GBM subject. NT-D regions in purple and TUM in pink. **H.** Plot representing ANCA (average number of copy number alterations) score for NT-D and TUM. Along the Y-axis is the ANCA score, along the x-axis is each GBM subject. NT-D regions in purple and TUM in pink. Significant difference observed in ANCA scores between the two groups (t(10) = −2.97, p = 0.014).

To identify tumor-specific chromosomal alterations, we analyzed matched tumor and non-tumor nuclei form the same individuals (Figure 4A). Canonical GBM aneuploidies, gain of chromosomes 7 and loss of chromosome 10, were detected in 30 to 40% of tumors (Figure 4B), consistent with reports (17), and were entirely absent from all matched NT nuclei. Although some NT and TUM samples exhibited similar overall proportions of aneuploid cells and comparable CIN scores (Figure 4C–G), tumors consistently displayed elevated ANCA scores (mean: 3.23 in TUM vs. 0.57 in NT; t(10) = −2.97, p = 0.014; 95% CI: −4.67 to −0.66; Figure 4H). These results confirm that tumor-specific aneuploidy patterns arise independently within the malignant compartment.

We also examined sex-chromosome alterations. Loss of X (LoX) was observed in 10.7% of all female nuclei (n=107), including one from a healthy control and nine from tumors. Tumor 4 showed the highest LoX burden (66%) despite having no autosomal aneuploidies in the analyzed nuclei, suggesting that X loss may contribute to tumor biology through distinct mechanisms. LoX was not observed in males. Gains of the chromosomes were detected exclusively on the Xp arm and were randomly distributed across groups, whereas LoX was restricted to female tumors (**Supplementary Figure 4**).

## Discussion

Aneuploidy is a hallmark of cancer, acting both as a driver of tumor evolution and as a consequence of disrupted chromosome segregation and broader genome instability. GBM exhibits high levels of chromosomal instability and complex structural alterations, features likely linked to poor prognosis and therapy resistance (17,60). Among canonical chromosomal alterations gain of chromosome 7 and loss of chromosome 10 are found in most tumors, but whether these imbalances arise *de novo* during transformation or reflect pre-existing chromosomal instability in phenotypically normal glial cells has remained unresolved. To address this question, we compared single nuclei profiles from NeuN-negative cortical nuclei in healthy, non-tumor, and tumor brain tissue and show that non-tumor glia harbor only low-frequency, mosaic aneuploidies, most notably involving chromosome 16p, while GBM-defining (+7/−10) are restricted to tumors. These findings indicate that CIN in GBM arises during malignant transformation rather than preceding it.

We found that 15.2% of glial nuclei from healthy individuals harbored detectable aneuploidies, predominantly affecting chromosome arms. This frequency is substantially higher than the 1–5% reported in postmitotic neurons by single-cell sequencing (20,41,44). The difference could reflect the proliferative capacity of glial progenitors, which remain mitotically active throughout adulthood and are therefore more vulnerable to replication errors, mitotic stress, and age-associated declines in DNA repair fidelity.

Technical factors also contribute. Our analytical pipeline distinguished between whole-chromosome and arm-level aneuploidies, enabling detection of rare or subclonal events that would be obscured in bulk CNV analyses. While metaphase karyotyping can resolve arm-level alterations at single-cell resolution, it requires actively dividing cells and is not feasible for most brain tissue. In contrast, snWGS profiles both dividing and non-dividing nuclei, allowing comprehensive assessment of genomic heterogeneity. By analyzing chromosome arms independently, we increased sensitivity to segmental events that might otherwise be missed. These methodological advances likely account for the higher frequencies we observed compared to neuron-focused studies (41,46,61) and, importantly, enabled identification of recurrent hotspots with potential biological relevance.

The types of aneuploidy we observed likely reflect distinct underlying mechanisms. Whole-chromosome aneuploidies arise primarily from mitotic segregation errors such as merotelic attachments, spindle checkpoint failure, or cohesion defects (62). In contrast, arm-level alterations are more consistent with structural lesions arising from replication stress, DNA double-strand breaks, or defective homologous recombination. Localized events such as breakage–fusion–bridge cycles or chromothripsis-like rearrangements may also produce focal arm-level changes, even in the absence of generalized CIN (63). Glial progenitors, which retain proliferative potential over decades, may gradually accumulate such lesion through oxidative stress, inflammation, or age-related declines in repair capacity.

The absence of increased CIN in normal glia may reflect the selective cost of GBM-defining alterations. The combined dosage effects of chromosome 7 gain and chromosome 10 loss amplify oncogenes (*EGFR*, *MET*, *CDK6*) while deleting tumor suppressors (*PTEN*, *MGMT*), changes that may overwhelm checkpoints and trigger senescence or apoptosis rather than clonal expansion. Such potent karyotypic imbalances likely become tolerable only after checkpoint attenuation or whole-genome duplication (WGD) provides buffering capacity (64). By contrast, lineage-specific aneuploidies in other cancers, such as chromosome 3q gain in squamous carcinomas, may exert weaker or more compatible selective effects, allowing them to persist in premalignant or even normal tissues.

These findings highlight a fundamental tissue-specific difference in the timing of chromosomal instability. In Barrett’s esophagus and colorectal adenomas, aneuploid clones emerge early and expand across histologically normal tissue, creating a field effect that predicts malignant progression defect (3,31). Similarly, squamous carcinomas often exhibit recurrent 3q gain in premalignant lesions (16). In contrast, our data show that non-tumor glia lack GBM-defining alterations such as +7/−10, instead displaying only simple, mosaic events such as 16p aneuploidy. This divergence suggests that gliomagenesis requires additional enabling events, such as checkpoint loss or WGD, before CIN can be tolerated and clonally selected.

These results also help contextualize the longstanding debate over somatic aneuploidy in the brain. Early interphase FISH studies reported aneuploidy rates as high as 5–12% in neurons, particularly involving chromosomes 17 and 21 (24,65). More recent single-cell genomic assays have challenged these estimates, attributing them to technical artifacts, hybridization noise, and limited genomic resolution (20,41). Our findings extend this discussion by showing that glial aneuploidy is neither rare nor random: it occurs at recurrent hotspots and is structurally distinct from both the relatively stable genomes of postmitotic neurons and the CIN-driven aneuploidy observed in GBM. By capturing both large-scale and focal alterations at single-nucleus resolution, this study redefines the baseline landscape of somatic structural variation in the adult human brain and establishes a necessary comparator for interpreting CIN in malignancy.

Among the most consistent findings across healthy and non-tumor glia we identified the recurrent alteration mapping to chromosome 16p11.2. This locus is flanked by large low-copy repeats (LCRs) and is highly susceptible to non-allelic homologous recombination (NAHR), which mediates recurrent deletions and duplications in both germline and somatic contexts (66). Unlike fragile sites such as FRA3B or FRA16D, which are prone to breakage under replication stress, 16p11.2 represents a sequence architecture–driven hotspot, where repetitive flanking sequences predispose the region to structural instability, independent of mitotic activity. Germline CNVs at 16p11.2 are among the most penetrant genetic risk factors for neurodevelopmental disorders, including autism spectrum disorder, schizophrenia, epilepsy, intellectual disability, and microcephaly (59,67,68). In our dataset, somatic deletions at this locus were detected in multiple individuals, with a shared 2.03 Mb overlapping region (chr16:14.8–16.8 Mb) encompassing approximately 30 protein-coding genes, including *NDE1*, a centrosomal protein required for mitotic spindle assembly and cortical neurogenesis (69), and NTA (N-terminal Asparagine Amidase), implicated in cognitive functions in animal models (70). Gene set enrichment analysis linked this recurrent region to the 16p13.11 CNV syndrome pathway (adj. p = 4.89 × 10^−^¹^9^), reinforcing its relevance to brain-related phenotypes. While 16p11.2 is well-known germline CNV hotspot, our findings demonstrate that it is also a site of recurrent somatic mosaicism in non-neoplastic glia, yet notably absent from GBM tumors. This suggests that 16p11.2 structural variation is tolerated in glia but not positively selected in gliomagenesis.

Mechanistically, 16p11.2 instability reflects a broader category of repeat-mediated genomic fragility. Although distinct from tandem repeat expansions such as *HTT*-CAG in Huntington’s disease (71) or C9orf72-GGGGCC in ALS/FTD (72), all forms of repeat-associated challenge replication fidelity, promote secondary structure formation, and interfere with recombination accuracy. In this context, 16p11.2 deletions highlight how non-coding architecture can drive somatic structural variation even in the absence of neoplastic selection.

Although the functional impact of somatic 16p11.2 deletions in glia remains undefined, their recurrence suggests they are not stochastic. Such mosaicism may contribute to interindividual variation in glial function, aging, or response to injury. Germline deletions at 16p11.2 have been associated with altered white matter integrity, reduced corpus callosum volume, and long-range connectivity disruptions (73), all phenotypes in which glia play key structural and regulatory roles. Whether somatic deletions can recapitulate similar effects is an open and testable hypothesis.

Finally, although 16p has previously been identified as a germline CNV hotspot associated with aging-related traits such as gait speed decline (74), its recurrent somatic involvement in the adult brain has not, to our knowledge, been previously reported. These findings suggest that CNV-prone loci may contribute to normal brain variation via post-zygotic, lineage-restricted genomic alterations, expanding our understanding of somatic mosaicism beyond cancer and congenital disease.

While somatic aneuploidy was clearly detected in non-tumor glial nuclei, its structure, frequency, and complexity were fundamentally distinct from the CIN observed in GBM tumors. Tumor nuclei exhibited significantly higher rates of whole-chromosome aneuploidy (p = 0.0023), a greater proportion of nuclei harboring both arm and chromosome-level alterations (p = 0.0003 vs. HCs; p = 0.0021 vs. NT), and elevated ANCA scores (3.23 in tumors vs. 0.57 in non-tumor tissue, p = 0.014). Canonical GBM events such as +7/−10 were present in 30–40% of tumor nuclei and absent from non-neoplastic glia.

These findings argue against a field cancerization model in GBM, in which adjacent cells acquire genomic priming that predisposes them to transformation. The absence of complex karyotypes and hallmark GBM alterations in non-tumor glia suggests that somatic aneuploidy in normal brain represents a distinct, constrained form of genomic variation.

The biological cost of aneuploidy is high: imbalanced karyotypes disrupt gene dosage, induce proteotoxic and metabolic stress, and activate p53-mediated checkpoints and innate immune sensing (75). In non-transformed cells, these stress responses typically trigger cell cycle arrest, senescence, or apoptosis (8,76). For CIN to arise and persist in GBM, tumor cells must overcome these intrinsic barriers, through attenuating DNA damage checkpoints, rewiring of proteostasis networks, or epigenetic silencing of stress-response programs.

A likely enabling event is whole-genome duplication (WGD), which is prevalent in GBM and associated with increased tolerance for chromosomal imbalance. WGD allows aneuploidy to occur in a polyploid background, buffering the impact of dosage changes and promoting CIN under selective pressure (64,77). In our study, WGD nuclei were significantly more likely to be aneuploid, and tumors with WGD exhibited higher ANCA scores and greater structural complexity, consistent with the hypothesis that WGD scaffolds the emergence of CIN (78).

Some non-tumor regions exhibited similar proportions of aneuploid nuclei as tumor regions. However, this numerical resemblance masked fundamental differences in karyotype architecture. Non-tumor glial nuclei typically harbored simple, isolated arm-level alterations affecting a single chromosome, whereas tumor nuclei displayed dense, multi-event profiles including canonical GBM imbalances, polyploid states, and co-occurring whole-chromosome and segmental alterations. This distinction reinforces the view that it is not merely the presence of aneuploidy, but its complexity and tolerance, that distinguishes malignant from non-malignant glial genomes.

Together, these findings suggest that CIN in GBM represents a discrete state shift rather than a gradual erosion of genomic integrity. This transition is likely gated by enabling events, including WGD and checkpoint suppression, that allow CIN to arise, persist, and evolve within the malignant compartment.

Beyond overall aneuploidy burden, our single-cell data revealed sex-specific patterns of chromosomal alterations in GBM tumors. Gain of chromosome 7p was more frequent in male tumors (53%) than female tumors (7%), while loss of 11p and 11q was enriched in female tumors (p = 0.0599 and 0.0475, respectively). These differences suggest that sex-specific selective pressures or genomic constraints shape karyotype evolution in gliomas, potentially reflecting variation in DNA repair capacity (38), immune surveillance (79), or hormone-regulated genome stability (80).

We also identified a female-specific pattern of X chromosome loss (LoX) in GBM tumors. LoX was detected in 10.7% of all female nuclei and absent from male tumors. In one tumor (GBM 4), LoX occurred in 66% of nuclei despite relative autosomal stability, suggesting that LoX may represent an alternative route to genomic imbalance in female gliomas. By contrast, X gains were rare, randomly distributed, and limited to Xp.

Loss of the Y chromosome (LoY) has been extensively studied in aging and male cancers, but LoX remains comparatively underexplored, particularly in gliomas. Our analysis revealed LoX in ~10% of female non-neoplastic glial nuclei, identifying a previously unrecognized form of somatic mosaicism in the adult brain. This aligns with large-scale studies showing that mosaic LoX is the most common clonal X-chromosome alteration in aging women and is associated with inherited risk variants and clonal selection (81). In females, approximately 15% of X-linked genes escape X-chromosome inactivation (82), thus LoX may result in haploinsufficiency of escapee genes, a mechanism not applicable to males, potentially influencing CIN tolerance and tumor evolution.

Mechanistically, LoX may affect GBM biology through multiple processes: (1) haploinsufficiency of X-linked tumor suppressors such as *KDM6A*, *ATRX*, or *FOXP3*, which regulate chromatin, DNA repair, and immune evasion; (2) loss of biallelic expression of escapee genes involved in immune signaling or proliferation (e.g., *TLR7*, *CD40LG*, *KDM5C*); (3) unmasking of recessive alleles on the remaining X chromosome; and (4) altered immune activation due to loss of X-linked immunoregulatory loci. LoX has been associated with CIN and poor prognosis in other cancers (83,84). Although its role in GBM remains to be defined, the presence of LoX in a genomically stable tumor suggests it may represent a non-canonical route to dysregulation, independent of classical CIN pathways.

In sum, this study defines the landscape of somatic aneuploidy in adult human glial cells and demonstrates that CIN in GBM is a transformation-associated process absent from surrounding non-neoplastic brain. By resolving arm-level and whole-chromosome alterations at single-nucleus resolution, we distinguish background glial mosaicism from the adaptive CIN that characterizes malignant evolution. The recurrent involvement of 16p11.2 highlights how genome architecture, particularly repeat-rich loci, can drive somatic structural diversity in normal cells, independent of neoplastic selection. In contrast, CIN in GBM is linked to enabling events such as WGD, checkpoint failure, and stress-pathway silencing. Finally, the discovery of sex-biased chromosomal alterations, including LoX, suggests that CIN pathways in glioma may be modulated by sex chromosome dosage, XCI escape, and sex-specific selective pressures. Future work should examine how somatic CNVs contribute to brain aging, plasticity, and disease, and whether sex-specific CIN mechanisms shape glioma evolution and therapeutic response. Integrating genomic, transcriptomic, and epigenetic data at single-cell resolution will be essential to connect somatic structural variation to cellular function in both normal and malignant brain compartments.

## Material and Methods

### Subject Cohorts and Tissue Collection and Processing

Post-mortem human brain tissues were obtained from the NIH NeuroBioBank (NBB)(85). All experimental procedures were approved by the Internal Review Board of the Albert Einstein College of Medicine (IRB #2018–9792). To establish a cohort of both GBM and healthy subjects, samples from the NBB were filtered to identify GBM patients with tumors preserved as frozen tissue based on experimental requirements. This process identified 6 GBM subjects (age: 63 ± 5.8 years; 3 females and 3 males). For each GBM subject, tissue was obtained from the tumor core and a non-tumor region distal from the tumor. To generate a control cohort, we selected age-matched healthy controls with brain regions matching, or as close as possible to, the non-tumor regions of the GBM cohort (age: 50.4 ± 12.4 years; 7 females and 5 males) (**Extended Data Table 1 and 7)**. Control samples were further filtered to exclude subjects with co-morbidities known to impact brain function (i.e., diabetes, Alzheimer’s). Approximately 100 mg of cryopreserved tissue was obtained for each subject. All tissues were reviewed a board-certified neuropathologist, Dr. Huttner, who confirmed the GBM diagnosis in the tumor specimens and verified that non-tumor tissues were free of tumor cells based on H&E staining. GBM tumors displayed hallmark histological features, including high cellularity, pleomorphism, microvascular proliferation, and pseudopalisading necrosis.

### Nuclei isolation

Nuclei isolation was adapted from Corces et al(86) and optimized for frozen brain tissues. While the original method used a dounce and pestle homogenizer, this approach left residual tissue fragments, limiting nuclei extraction. To address this, tissue dissociation was performed using the POLYTRON PT 1200 E (Kinematica, 11010025 Lucerne, Switzerland) which improved efficiency. A 2 mm biopsy punch core (Integra 33–31-P/25, Preston NJ) was used to collect tissue fragments from the frozen brain, which were transferred to a 50 mL tube containing 2 mL of cold 1x Homogenization Buffer (sucrose, EDTA, 10% NP-40, PMSF and β-mercaptoethanol) and allowed to thaw on ice for approximately five minutes. Tissues were homogenized using the POLYTRON PT 1200 E set at half speed for 1.5 minutes on ice. An Iodixanol (Sigma D1556–250ML, St Luis MO) gradient was prepared in a 15 mL tube by layering a 29% Iodixanol solution (sucrose, PMSF, β-mercaptoethanol, CaCl2, Mg (Ac)2 and Tris pH7.8) over a 35% iodixanol solution containing the same reagents. The brain homogenate was mixed with a 50% Iodixanol solution (containing the same reagents) in a 1:1 ratio and layered on top the 29% solution. Clear separation of layers was observed at 3 mL and 6 mL within the 15 mL tube, indicating the formation of a gradient. The gradient was centrifuged at 3,000 RCF without brake for 20 min at 4°C in a pre-chilled centrifuge (ThermoFisher 75009509, Waltham MA). After centrifugation, nuclei were isolated from the interface between the 29% and 35% layers and carefully collected for downstream experiments.

### FACS sorting of cortical NeuN-negative single nuclei

Isolated nuclei were incubated in PBS containing 10% goat serum (ThermoFisher 50197Z, Waltham MA) for 1 hour on ice to block non-specific binding. After centrifugation at 4,000 RPM for 10 minutes, the nuclei were resuspended in PBS containing NeuN Alexa-488 antibody (ThermoFisher MAB377X, Waltham MA) at a 1:500 dilution and kept on ice for 1 hour. Following this, nuclei were then spun down again, resuspended in PBS with DRAQ5 DNA dye (eBioscience 65-0880-92, San Diego CA) at a 1:500 dilution, filtered through a sterile CellTrics 20 mm disposable filter (Sysmex 04-004-2325, Lincolnshire IL) into a 5 mL Polystyrene Round-Bottom tube (Corning Falcon 352058, Corning NY), and kept on ice until sorting. NeuN-neg DRAQ5+ single nuclei were sorted Using the MoFlo Astrios Cell Sorter equipped with a 100 mn nozzle with a sheath pressure of 25 psi, into 12 PCR Tube Strips 0.1 mL + Cap strips, flat bottom (Eppendorf 0030124820, Hamburg, Germany), each containing 3 mL of PBS. Following isolation into single PCR strips, nuclei were capped, centrifuged, immediately placed onto dry ice, and stored at −80 °C until further use. Prior to FACS, antibody dilutions were optimized using varying concentrations and nuclei were dropped on slides for visualization under a fluorescence microscope to confirm specific immunofluorescence staining.

### Ultra-low coverage single nuclei whole genome sequencing

Single nuclei libraries were generated for single NeuN-neg nuclei using the PicoPLEX Gold Single Cell DNA-seq kit (Takara R300669, San Jose CA), and indexed using DNA single index Kit (Takara R400660, San Jose CA). To minimize PCR-induced errors, amplification cycles were limited to 8. Library quality (size of DNA fragments and regional molarity) was assessed via Tapestation, and those with a wide peak centered around 800bps and a regional molarity greater than 100 pmol/l were deemed suitable for sequencing. Sequencing was performed on NextSeq500 in the Genomics Center at Rutgers New Jersey Medical School using the 2×150 bp sequencing mode. Raw sequence reads were adapter- and quality-trimmed using Trim Galore (version 0.4.1) and aligned to GRCh37-hg19 using BWA MEM (version 0.7.13) (87). PCR duplications were removed using samtools (version 0.1.19)(88) Reads were realigned around the known INDELs, and base qualities were recalibrated based on known SNPs with GenomeAnalysisTK (version 3.5) (https://gatk.broadinstitute.org/hc/en-us). The reads with mapping quality above 30 were kept for downstream analysis. Aligned bam files were converted to bed files using bamToBed (version 2.30.0)(89). Ginkgo was used for identifying copy number variations, using 500K bin size and all other settings as default(90).

Control samples, including a euploid (Coriell GM12878, Camden NJ) and a Trisomy 21 cell line (Coriell AG08942, Camden NJ) were sequenced in each sequencing round to verify sequencing accuracy and establish a baseline noise inherent to this single-cell sequencing approach.

Sequencing coverage was assessed by calculating the proportion of bases covered for each chromosome relative to chromosome length. Uniformity of coverage was verified before analysis. Regions with reduced coverage due to sequencing challenges, such as telomeres, centromeres, and GC-rich regions, were blacklisted as low-confidence regions and excluded from further analysis.

### Statistical Analyses

One-way ANOVA was used to assess significance on bar charts comparing aneuploidy frequencies across multiple cohort groups, while t-test was used for comparison between two groups. For bubble plots, Euclidean distance measurements were calculated, and one-way ANOVA was performed to compare the distances across the cohort groups.

### Simulation-Based Threshold Determination for Significant Deviations in Chromosome Copy Number Variation

To estimate expected non-diploid events per chromosome in each cohort, we performed 10,000 simulations using the number of analyzed nuclei for each group, assuming each nucleus contained 38 chromosomes and chromosome arms. The set.seed(123) function in R was used for reproducibility of random number generation. Non-diploid counts were simulated using a binomial distribution, with the probability of success set to 1/38 per chromosome. A significance threshold, set at the 90^th^ percentile of the simulated counts, was used to identify chromosomes with significant deviations. Chromosome counts were reshaped using the pivot_longer function from the tidyr package for individual chromosome analysis. The data was grouped by chromosome, and counts were calculated based on whether the chromosome count was equal to 2 (diploid) or not. After summarization, the grouping was removed, and total counts were compared against the established threshold.

### Group comparison

Healthy controls were classified based on their chromosomal status into euploid and aneuploid groups based on the counts of aneuploid cells in 2n and 3n categories, and the number of affected chromosomes. Subjects were designated as euploid if they had no aneuploidy detected in any of the measured categories (Arm-2n = 0, Arm & Chr. 2n = 0, Arm-3n = 0, Arm & Chr. 3n = 0 and 3n = 0); otherwise, they were classified as aneuploid. The Mann-Whitney U test (Wilcoxon rank sum test) was performed to assess differences between the euploid and aneuploid groups for each relevant variable, employing normal approximation for cases with ties. Statistical analyses were conducted using R version [RStudio 2024.09.0], and a significance level of p < 0.05 was considered statistically significant.

### Gains versus losses NT and TUM

We evaluated chromosomal gains, losses, and no copy number change across 96 single cells (51NT and 45TUM) spanning 38 chromosomes and chromosome arms. Each segment was classified as gain, loss, or no change based on deviations from the modal chromosomal copy number (2n). To determine whether gains or losses were more likely to occur, we performed an exact binomial test on the total counts of gains and losses across all nuclei. The null hypothesis assumed an equal probability (50%) for gains and losses. Out of 3,648 total informative events (excluding unchanged segments), 309 were gains and 71 were losses, with 3,268 segments showing no change. The test was performed using the binom.test() function in R, with the alternative hypothesis that the probability of gain differs from 0.5.

## Supplementary Material

Supplementary Files

This is a list of supplementary files associated with this preprint. Click to download.

- Albert.et.alSupplementaryMaterial92525.docx
- SupplementaryFigureSINGLECELLPROFILESALLSAMPLES.pdf
- RS1128.pdf

## Figures and Tables

**Table 1:** Demographic and Clinical Characteristics of Healthy Control Subjects.

Sample ID	Clinical Brain Diagnosis	Sex	Age (years)	Brain Area T/NT
**HC1**	No clinical brain diagnosis found	Male	43	BA5
**HC2**	No clinical brain diagnosis found	Male	35	BA4
**HC3**	No clinical brain diagnosis found	Male	37	BA8
**HC4**	No clinical brain diagnosis found	Male	37	BA8
**HC5**	No clinical brain diagnosis found	Male	44	BA8
**HC6**	No clinical brain diagnosis found	Female	40	BA4
**HC7**	No clinical brain diagnosis found	Female	65	BA4
**HC8**	No clinical brain diagnosis found	Male	58	BA4
**HC9**	No clinical brain diagnosis found	Male	63	BA8
**HC10**	No clinical brain diagnosis found	Female	59	BA5
**HC11**	No clinical brain diagnosis found	Female	68	BA8
**HC12**	No clinical brain diagnosis found	Female	73	BA4

HC= Healthy Controls; BA=Brodmann Area

**Table 2:** Demographic and Clinical Characteristics of High-Grade Glioma (GBM) Subjects.

Sample ID	Clinical Brain Diagnosis	Sex	Age (years)	Brain Area T/NT
**TUM1/NT1**	Glioblastoma (Grade IV)	Male	66	BA22/BA8
**TUM2/NT2**	Glioblastoma (Grade IV)	Male	59	BA44/BA4
**TUM3/NT3**	Glioblastoma (Grade IV)	Male	62	White Matter/BA8
**TUM4/NT4**	Glioblastoma (Grade IV)	Female	55	Corpus/BA5
**TUM5/NT5**	Glioblastoma (Grade IV)	Female	67	BA31/BA8
**TUM6/NT6**	Glioblastoma (Grade IV)	Female	71	BA31/BA8

TUM= Tumor; NT=Non-tumor; BA=Brodmann Area

## Data Availability

Raw sequencing data (FASTQ files) have been uploaded to The NeuroBioBank Data Repository within the NIMH Data Archive (NDA) under project ID C5801 “Whole-Genome Sequence Analysis of Postmortem Human Brains from the NIH NeuroBioBank”. The data will be made available upon acceptance of the manuscript. Additionally, metadata associated with the dataset, including (e.g., sample information, gender, disease state), can be accessed through the same repository.

## Statement of Significance

This study establishes the baseline somatic aneuploidy landscape in adult glia, identifies 16p as a recurrent non-tumor variation, and demonstrates that GBM-defining chromosomal instability arises only during malignant transformation.
